# Supplementary material for: Pure spin photocurrent in non-centrosymmetric crystals: bulk spin photovoltaic effect
Source: Nat Commun. 2021 Jul 15;12:4330. doi: 10.1038/s41467-021-24541-7 (PMC8282873; doi:10.1038/s41467-021-24541-7)
Supplement: Supplementary file 1 — Supplementary Information [file 41467_2021_24541_MOESM1_ESM.pdf]

Supplementary Materials
to
Pure Spin Photocurrent in Non-centrosymmetric Crystals: Bulk Spin Photovoltaic Effect

Haowei Xu[1], Hua Wang[1], Jian Zhou[1], and Ju Li[1,2]

[1]Department of Nuclear Science and Engineering, Massachusetts Institute of Technology, Cambridge, Massachusetts 02139, USA

[1]Department of Materials Science and Engineering, Massachusetts Institute of Technology, Cambridge, Massachusetts 02139, USA

Table of Contents

1 Derivations of NLO spin current conductivity **2**

2 Reproduce the shift and injection charge current formulae **6**

2.1 NLO charge conductivities in the length gauge 6

2.2 Equivalence between the velocity gauge and the length gauge 8

3 Shift- and injection-like mechanisms for the spin current **10**

3.1 $\mathcal{P}$-broken, $\mathcal{T}$-conserved systems 10

3.2 $\mathcal{P}$-broken, $\mathcal{T}$-broken, $\mathcal{PT}$-conserved systems 11

3.3 $\mathcal{P}$-broken, $\mathcal{T}$-broken, $\mathcal{PT}$-broken system 11

4 The definition of spin current **12**

5 Experimental considerations **13**

5.1 Estimation of the temperature rise 13

5.2 Maximum spin current density 14

5.3 Detection of the spin current 15

6 Computational benchmarks **15**

6.1 Numerical comparison between the velocity gauge and length gauge 16

6.2 NLO conductivity as a function of lifetime τ 16

6.3 NLO conductivity as a function of SOC strength 18

7 NLO spin current under circularly polarized light **19**

1 Derivations of NLO spin current conductivity

In this section we derive the NLO spin current conductivity from quadratic response theory, in a similar fashion to Refs. [1, 2]. The Hamiltonian of the system can be written as

$$H = H_0 + V \tag{S1}$$

where H_0 is the unperturbed Hamiltonian, while V is a perturbation, from e.g. the interaction between light and the electrons. Without the interaction term V, the equilibrium density matrix should be

$$\rho_0 = \frac{1}{Z} e^{-\beta H_0} \tag{S2}$$

Note that $[\rho_0, H_0] = 0$. When V is turned on, the equation of motion of the density matrix $\rho(t)$ should be (von Neumann equation)

$$\frac{\partial \rho}{\partial t} = -\frac{i}{\hbar}[H, \rho] - \frac{\rho - \rho_0}{\tau} \tag{S3}$$

The last term $-\frac{\rho-\rho_0}{\tau}$ is a dissipation that describes the interaction between the system and the heat bath: the system always has the tendency to return to ρ_0. Here we adopted the constant relaxation time approximation and use a uniform τ for all states. In practice, τ should be different for different states (e.g., electrons and holes may have different τ). More rigorously, one should use $\frac{\partial \rho}{\partial t}|_{\mathrm{col}}$, which

incorporates all relaxation processes, such the scattering of the electrons/holes with phonons, impurities, etc, and the spin relaxation process. But we made simplifications by using $\frac{\partial\rho}{\partial t}|_{\rm col} \approx -\frac{\rho-\rho_0}{\tau}$.

In order to solve Eq. (S3), we use a trick similar to the transformation between the Schrodinger picture and the interaction picture. Let $\tilde{\rho}(t) = e^{\frac{t}{\tau}} e^{i\frac{H_0}{\hbar}t}\rho(t)e^{-i\frac{H_0}{\hbar}t}$, then we have

$$
\begin{aligned}
\frac{\partial\tilde{\rho}}{\partial t} &= \frac{1}{\tau}e^{\frac{t}{\tau}}e^{i\frac{H_0}{\hbar}t}\rho(t)e^{-i\frac{H_0}{\hbar}t} + i\frac{H_0}{\hbar}e^{\frac{t}{\tau}}e^{i\frac{H_0}{\hbar}t}\rho(t)e^{-i\frac{H_0}{\hbar}t} + e^{\frac{t}{\tau}}e^{i\frac{H_0}{\hbar}t}\frac{\partial\rho(t)}{\partial t}e^{-i\frac{H_0}{\hbar}t} + e^{\frac{t}{\tau}}e^{i\frac{H_0}{\hbar}t}\rho(t)(-i\frac{H_0}{\hbar})e^{-i\frac{H_0}{\hbar}t} \\
&= e^{\frac{t}{\tau}}e^{i\frac{H_0}{\hbar}t}\left\{\frac{\rho}{\tau} + \frac{i}{\hbar}[H_0,\rho] - \frac{i}{\hbar}[H,\rho] - \frac{\rho-\rho_0}{\tau}\right\}e^{-i\frac{H_0}{\hbar}t} \\
&= -\frac{i}{\hbar}[\tilde{V},\tilde{\rho}] + \frac{\rho_0}{\tau}e^{\frac{t}{\tau}}
\end{aligned}
\tag{S4}
$$

where $\tilde{V}(t) = e^{i\frac{H_0}{\hbar}t}V(t)e^{-i\frac{H_0}{\hbar}t}$. Then we can integrate Eq. (S4) to get

$$
\begin{aligned}
\tilde{\rho}(t) &= \tilde{\rho}(0) - \frac{i}{\hbar}\int_0^t \mathrm{d}t'[\tilde{V}(t'),\tilde{\rho}(t')] + \frac{\rho_0}{\tau}\int_0^t \mathrm{d}t' e^{\frac{t'}{\tau}} \\
&= \rho_0 + \rho_0\left(e^{\frac{t}{\tau}} - 1\right) - \frac{i}{\hbar}\int_0^t \mathrm{d}t'[\tilde{V}(t'),\tilde{\rho}(t')] \\
&= \rho_0 e^{\frac{t}{\tau}} - \frac{i}{\hbar}\int_0^t \mathrm{d}t'[\tilde{V}(t'),\tilde{\rho}(t')] \\
&= \rho_0 e^{\frac{t}{\tau}} - \frac{i}{\hbar}\int_0^t \mathrm{d}t'\left[\tilde{V}(t'), \rho_0 e^{\frac{t'}{\tau}} - \frac{i}{\hbar}\int_0^{t'} \mathrm{d}t''[\tilde{V}(t''),\tilde{\rho}(t'')]\right] \\
&= \rho_0 e^{\frac{t}{\tau}} - \frac{i}{\hbar}\int_0^t \mathrm{d}t'[\tilde{V}(t'),\rho_0 e^{\frac{t'}{\tau}}] - \frac{i}{\hbar}\int_0^t \mathrm{d}t'\left[\tilde{V}(t'), -\frac{i}{\hbar}\int_0^{t'} \mathrm{d}t''[\tilde{V}(t''),\tilde{\rho}(t'')]\right] \\
&= \cdots
\end{aligned}
\tag{S5}
$$

By iteratively putting $\tilde{\rho}(t)$ into the bracket on the rightmost of the equation above, we can obtain

$$
\tilde{\rho}(t) = \tilde{\rho}^{(0)}(t) + \tilde{\rho}^{(1)}(t) + \tilde{\rho}^{(2)}(t) + \cdots \tag{S6}
$$

with

$$
\begin{aligned}
\tilde{\rho}^{(0)}(t) &= \rho_0 e^{\frac{t}{\tau}} \\
\tilde{\rho}^{(n+1)}(t) &= -\frac{i}{\hbar}\int_0^t \mathrm{d}t'[\tilde{V}(t'),\tilde{\rho}^{(n)}(t')]
\end{aligned}
\tag{S7}
$$

Then going back from $\tilde{\rho}(t)$ to $\rho(t)$ with $\rho(t) = e^{-\frac{t}{\tau}}e^{-i\frac{H_0}{\hbar}t}\tilde{\rho}(t)e^{i\frac{H_0}{\hbar}t}$, we have

$$
\rho(t) = \rho^{(0)}(t) + \rho^{(1)}(t) + \rho^{(2)}(t) + \cdots \tag{S8}
$$

with

$$
\rho^{(0)} = \rho_0 \tag{S9}
$$

and

$$\begin{aligned}
\rho^{(n+1)}(t) &= e^{-\frac{t}{\tau}}e^{-i\frac{H_0}{\hbar}t}\tilde{\rho}^{(n+1)}(t)e^{i\frac{H_0}{\hbar}t} \\
&= -\frac{i}{\hbar}\int_0^t \mathrm{d}t' e^{-\frac{t}{\tau}}e^{-i\frac{H_0}{\hbar}t}[\tilde{V}(t'),\tilde{\rho}^{(n)}(t')]e^{i\frac{H_0}{\hbar}t} \\
&= -\frac{i}{\hbar}\int_0^t \mathrm{d}t' e^{-\frac{t-t'}{\tau}}e^{-i\frac{H_0}{\hbar}(t-t')}[V(t'),\rho^{(n)}(t')]e^{i\frac{H_0}{\hbar}(t-t')} \\
&= \frac{i}{\hbar}\int_0^t \mathrm{d}t' e^{-\frac{t'}{\tau}}e^{-i\frac{H_0}{\hbar}t'}[V(t-t'),\rho^{(n)}(t-t')]e^{i\frac{H_0}{\hbar}t'}
\end{aligned} \tag{S10}$$

Next we expand $V(t-t')$ with a Fourier transformation $V(t-t') = \int \frac{\mathrm{d}\omega}{2\pi}V(\omega)e^{i\omega(t-t')}$. Then $\rho^{(1)}$ can be calculated as

$$\begin{aligned}
\rho^{(1)}_{nm}(t) &= \langle n|\rho^{(1)}(t)|m\rangle \\
&= \frac{i}{\hbar}\int_0^t \mathrm{d}t' \left\langle n\middle| e^{-\frac{t'}{\tau}}e^{-i\frac{H_0}{\hbar}t'}[V(t-t'),\rho_0]e^{i\frac{H_0}{\hbar}t'}\middle| m\right\rangle \\
&= \frac{i}{\hbar}\int \frac{\mathrm{d}\omega}{2\pi}\langle n|[V(\omega),\rho_0]|m\rangle e^{i\omega t}\int_0^t \mathrm{d}t \exp\left(\frac{i}{\hbar}\left[(E_m-E_n)+\frac{i\hbar}{\tau}-\hbar\omega\right]t'\right) \\
&= \frac{i}{\hbar}\int \frac{\mathrm{d}\omega}{2\pi}V_{nm}(\omega)(f_{mm}-f_{nn})e^{i\omega t}\frac{\exp\left(\frac{i}{\hbar}\left[(E_m-E_n)+\frac{i\hbar}{\tau}-\hbar\omega\right]t\right)-1}{\frac{i}{\hbar}\left[(E_m-E_n)+\frac{i\hbar}{\tau}-\hbar\omega\right]} \\
&= \int \frac{\mathrm{d}\omega}{2\pi}e^{i\omega t}\frac{f_{nm}V_{nm}(\omega)}{E_{mn}-\hbar\omega+\frac{i\hbar}{\tau}}
\end{aligned} \tag{S11}$$

Obviously,

$$\rho^{(1)}_{nm}(\omega;\omega) = \frac{f_{nm}V_{nm}(\omega)}{E_{mn}-\hbar\omega+\frac{i\hbar}{\tau}} \tag{S12}$$

Then, the second order $\rho^{(2)}$

$$\begin{aligned}
\rho^{(2)}_{nm}(t) &= \langle n|\rho^{(2)}(t)|m\rangle \\
&= \frac{i}{\hbar}\int \frac{\mathrm{d}\omega'}{2\pi}e^{i\omega t}\int_0^t \mathrm{d}t' \exp\left(\frac{i}{\hbar}\left[(E_m-E_n)+\frac{i\hbar}{\tau}-\hbar\omega'\right]t'\right)\sum_l \left(V_{nl}(\omega')\rho^{(1)}_{lm}(t-t')-\rho^{(1)}_{nl}(t-t')V_{lm}(\omega')\right) \\
&= \int \frac{\mathrm{d}\omega}{2\pi}\int \frac{\mathrm{d}\omega'}{2\pi}e^{i(\omega+\omega')t}\frac{1}{E_{mn}-\hbar(\omega+\omega')+\frac{i\hbar}{\tau}}\sum_l\left(\frac{f_{lm}V_{nl}(\omega')V_{lm}(\omega)}{E_{ml}-\hbar\omega+\frac{i\hbar}{\tau}}-\frac{f_{nl}V_{nl}(\omega)V_{lm}(\omega')}{E_{ln}-\hbar\omega+\frac{i\hbar}{\tau}}\right)
\end{aligned} \tag{S13}$$

We have

$$\rho^{(2)}_{nm}(\omega+\omega';\omega,\omega') = \frac{1}{E_{mn}-\hbar(\omega+\omega')+\frac{i\hbar}{\tau}}\sum_l\left(\frac{f_{lm}V_{nl}(\omega')V_{lm}(\omega)}{E_{ml}-\hbar\omega+\frac{i\hbar}{\tau}}-\frac{f_{nl}V_{nl}(\omega)V_{lm}(\omega')}{E_{ln}-\hbar\omega+\frac{i\hbar}{\tau}}\right) \tag{S14}$$

For an arbitrary operator θ, the thermal expectation value of θ should be

$$\langle\theta\rangle = \mathrm{Tr}(\theta\rho) \tag{S15}$$

The first order response is

$$\begin{aligned}\langle\theta\rangle^{(1)}(\omega;\omega) &= \int \frac{\mathrm{d}\boldsymbol{k}}{(2\pi)^3}\sum_{mn}\theta_{mn}\rho^{(1)}_{nm}(\omega;\omega)\\ &= \int \frac{\mathrm{d}\boldsymbol{k}}{(2\pi)^3}\sum_{mn}\frac{f_{nm}\theta_{mn}V_{nm}(\omega)}{E_{nm}-\hbar\omega+\frac{i\hbar}{\tau}}\end{aligned} \tag{S16}$$

The the second order response is

$$\begin{aligned}\langle\theta\rangle^{(2)}(\omega+\omega';\omega,\omega') &= \int \frac{\mathrm{d}\boldsymbol{k}}{(2\pi)^3}\sum_{mn}\theta_{mn}\rho^{(2)}_{nm}(\omega+\omega';\omega,\omega)\\ &= \int \frac{\mathrm{d}\boldsymbol{k}}{(2\pi)^3}\sum_{mnl}\frac{\theta_{mn}}{E_{mn}-\hbar(\omega+\omega')+\frac{i\hbar}{\tau}}\left(\frac{f_{lm}V_{nl}(\omega')V_{lm}(\omega)}{E_{ml}-\hbar\omega+\frac{i\hbar}{\tau}}-\frac{f_{nl}V_{nl}(\omega)V_{lm}(\omega')}{E_{ln}-\hbar\omega+\frac{i\hbar}{\tau}}\right)\\ &= \int \frac{\mathrm{d}\boldsymbol{k}}{(2\pi)^3}\sum_{mnl}\frac{f_{lm}V_{lm}(\omega)}{E_{ml}-\hbar\omega+i\frac{\hbar}{\tau}}\left(\frac{\theta_{mn}V_{nl}(\omega')}{E_{mn}-\hbar(\omega+\omega')+i\frac{\hbar}{\tau}}-\frac{V_{mn}(\omega')\theta_{nl}}{E_{nl}-\hbar(\omega+\omega')+i\frac{\hbar}{\tau}}\right)\end{aligned} \tag{S17}$$

The last equity can be obtained with an interchange of dummy variables as $(n\to l, l\to m, m\to n)$. For the nonlinear spin current effect, the interaction is

$$\begin{aligned}V(\omega) &= -e\sum_{i=1}^{N}\boldsymbol{v}_i\cdot\boldsymbol{A}(\omega)\\ &= \frac{ie}{\omega}\sum_{i=1}^{N}\boldsymbol{v}_i\cdot\boldsymbol{\mathcal{E}}(\omega)\end{aligned} \tag{S18}$$

where $\boldsymbol{A}$ is the vector potential while $\boldsymbol{\mathcal{E}}$ is the electric field. After a second quantization, we have $V_{nm}(\omega)$ in the basis of Bloch waves

$$V_{nm}(\omega) = \frac{ie}{\omega}v^b_{nm}\mathcal{E}_b(\omega) \tag{S19}$$

For the spin current in the a direction with spin component i, we should have $\theta_{nm} = j^{a,i}_{nm} = \frac{1}{2}\{v^a, s^i\}_{nm}$, where $s^i = \frac{\hbar}{2}\sigma^i$ is the spin operator.

Finally, the nonlinear direct spin current is

$$\langle j^{a,s^i}\rangle^{(2)}(0;\omega,-\omega) = -\frac{e^2}{\omega^2}\int\frac{\mathrm{d}\boldsymbol{k}}{(2\pi)^3}\sum_{mnl}\frac{f_{lm}v^b_{lm}}{E_{ml}-\hbar\omega+i\frac{\hbar}{\tau}}\left(\frac{j^{a,s^i}_{mn}v^c_{nl}}{E_{mn}+i\frac{\hbar}{\tau}}-\frac{v^c_{mn}j^{a,s^i}_{nl}}{E_{nl}+i\frac{\hbar}{\tau}}\right)\mathcal{E}_b(\omega)\mathcal{E}_c(-\omega) \tag{S20}$$

And the spin conductivity can be written as

$$\sigma^{a,s^i}_{bc}(0;\omega,-\omega) = -\frac{e^2}{\hbar^2\omega^2}\int\frac{\mathrm{d}\boldsymbol{k}}{(2\pi)^3}\sum_{mnl}\frac{f_{lm}v^b_{lm}}{\omega_{ml}-\omega+\frac{i}{\tau}}\left(\frac{j^{a,s^i}_{mn}v^c_{nl}}{\omega_{mn}+\frac{i}{\tau}}-\frac{v^c_{mn}j^{a,s^i}_{nl}}{\omega_{nl}+\frac{i}{\tau}}\right) \tag{S21}$$

where we have replaced E_{mn} with $\hbar\omega_{mn}$. Eq. (S21) is exactly the same as Eq. (2) in the main text.

2 Reproduce the shift and injection charge current formulae

2.1 NLO charge conductivities in the length gauge

In Sec. 1, we adopted the so-called *velocity* gauge in deriving the NLO spin/charge conductivity, where the interaction with the oscillating electric field is described by the vector potential and velocity as in Eq. (S18). A charge current version of Eq. (S21), where one sets $j^{a,s^0} = ev^a$

$$\sigma^a_{bc}(0;\omega,-\omega) = -\frac{e^3}{\hbar^2\omega^2}\int\frac{\mathrm{d}\boldsymbol{k}}{(2\pi)^3}\sum_{mnl}\frac{f_{lm}v^b_{lm}}{\omega_{ml}-\omega+\frac{i}{\tau}}\left(\frac{v^a_{mn}v^c_{nl}}{\omega_{mn}+\frac{i}{\tau}}-\frac{v^c_{mn}v^a_{nl}}{\omega_{nl}+\frac{i}{\tau}}\right) \tag{S22}$$

has been derived in Refs. [1, 2] and has been used in e.g. Refs. [3, 4]. On the other hand, a different version of the formulae [5] for calculating the NLO charge conductivity, which uses the *length* gauge, seems more popular in literatures. In the length gauge, the interaction with light is described by $V = -e\boldsymbol{r}\cdot\boldsymbol{\mathcal{E}}$, where r is the position (length) operator. We first give a brief description on the formulae in length gauge, and then show that they are equivalent with our Eq. (S22).

In a time reversal symmetric system, the NLO charge current is divided into two parts, namely the *shift* current and the *injection* current,

$$\begin{aligned} j^a_{\text{shift}} &= 2\zeta^a_{bc}(0;\omega,-\omega)E^b(\omega)E^c(-\omega) \\ \frac{\mathrm{d}j^a_{\text{circular}}}{\mathrm{d}t} &= 2\eta^a_{bc}(0;\omega,-\omega)E^b(\omega)E^c(-\omega) \end{aligned} \tag{S23}$$

The shift current conductivity is

$$\zeta^a_{bc}(0;\omega,-\omega) = -\frac{i\pi e^3}{2\hbar^2}\int_{BZ}\frac{\mathrm{d}^3\boldsymbol{k}}{(2\pi)^3}\sum_{n,m}f_{nm}(r^b_{mn}r^c_{nm;a}+r^c_{mn}r^b_{nm;a})\delta(\omega_{mn}-\omega) \tag{S24}$$

where

$$r^a_{nm;b} = \frac{\partial r^a_{nm}}{\partial k^b} - i[\xi^b_{nn}-\xi^b_{mm}]r^a_{nm} \tag{S25}$$

is the generalized derivative of position operator r with respect to k and $\xi^a_{nn} = i\langle u_{nk}|\partial_{k^a}|u_{nk}\rangle$, where $|u_{nk}\rangle$ is the cell-periodic part of the wavefunction $|nk\rangle$. Note that for Bloch waves, it is not straightforward to define the full matrix of the position operator $r_{mn} = \langle m|r|n\rangle$, since the Bloch waves are infinite

in space. Usually r_{mn} is defined with only the interband elements

$$r_{mn} = \begin{cases} \dfrac{v_{mn}}{i\omega_{mn}}, & m \neq n \\ 0, & m = n \end{cases} \tag{S26}$$

But the definition of ξ is valid for intraband elements with $m = n$ as well.

Under a linearly polarized light $b = c$, Eq. (S24) can be further simplified as

$$\sigma^a_{bb}(0;\omega,-\omega) = -\frac{\pi e^3}{2\hbar^2}\int_{BZ}\frac{\mathrm{d}^3\boldsymbol{k}}{(2\pi)^3}\sum_{n,m} f_{nm}R^a_{nm}|r^b_{nm}|^2\delta(\omega_{mn}-\omega) \tag{S27}$$

where

$$r^b_{nm} = |r^b_{nm}|e^{-i\phi_{nm}}$$

and

$$R^a_{nm} = \frac{\partial\phi_{nm}}{\partial k^a} + \xi^a_{nn} - \xi^a_{mm}$$

is called the *shift vector*.

The injection current response function is

$$\eta^a_{bc}(0;\omega,-\omega) = \frac{\pi e^3}{2\hbar^2}\int_{BZ}\frac{\mathrm{d}^3\boldsymbol{k}}{(2\pi)^3}\sum_{n,m} f_{nm}\Delta^a_{nm}[r^c_{mn}, r^b_{nm}]\delta(\omega_{mn}-\omega) \tag{S28}$$

where $\Delta^a_{nm} = v^a_{nn} - v^a_{mm}$ and $[r^c_{mn}, r^b_{nm}] = r^c_{mn}r^b_{nm} - r^b_{mn}r^c_{nm}$.

The shift current can be induced by a linearly polarized light ($b = c$), and is a static current. On the other hand, the injection current cannot be induced by a linearly polarized light, and it should grow with time, thus it is somewhat like *injected* into the system. If the carrier lifetime is τ, then there should be a dissipation term $-j_{\text{injection}}/\tau$. And in the static limit ($t \to \infty$),

$$j_{\text{injection}} = 2\tau\eta^a_{bc}(0;\omega,-\omega)E^b(\omega)E^c(-\omega) \tag{S29}$$

so the *effective* conductivity should be defined as $\tau\eta^a_{bc}(0;\omega,-\omega)$. Eqs. (S24, S28) are derived in Ref. [5] and is widely used in many papers.

Here we would like the remark that the physical mechanism of the shift current and injection current are more evident in the length gauge format. Eqs. (S27, S28) are reminiscent of the Fermi's golden rule. The Dirac functions indicate that light induces resonant interband transitions, and the transition rate are proportional to $|r^b_{mn}|^2$ for LPL, and $[r^c_{mn}, r^b_{nm}]$ for CPL. Then the shift current comes from the fact that the wavefunction centers of electrons and holes differ by a factor of R^a_{nm}, which leads to a electric dipole eR^a_{nm}. The time derivative of the electric dipole is the current. On the other hand,

the injection current comes from the fact that the electrons and holes have different velocities, and the net current is determined by the velocity difference $\Delta^a_{nm} = v^a_{nn} - v^a_{mm}$.

2.2 Equivalence between the velocity gauge and the length gauge

Eq. (S22) and Eqs. (S24, S28) look very different, but they should give the same result in cases that they are both applicable, because they are dealing with the same physical problem: the charge current generated under light illumination. The difference is that, Eq. (S22) uses the velocity gauge and the light coupling with the system is included by replacing $\boldsymbol{p}$ with $\boldsymbol{p} - \frac{e}{c}\boldsymbol{A}$, where $\boldsymbol{p}$ is the momentum operator and $\boldsymbol{A}$ is the vector potential. Whereas Eqs. (S24, S28) use the length gauge and the light interaction is included by an additional term in the Hamiltonian $H_{\text{int}} = -e\boldsymbol{r}\cdot\boldsymbol{E}$, where $\boldsymbol{r}$ is the position operator and $\boldsymbol{E}$ is the electric field. These approaches should be equivalent and lead to the same results, as discussed in e.g. Refs. [6, 7].

Both velocity gauge and length gauge are applicable regardless time reversal symmetry $\mathcal{T}$. However, in deriving Eqs. (S24, S28), Ref. [5] assumed a $\mathcal{T}$-conserved system and made some simplifications. Thus Eqs. (S24, S28) are not valid when $\mathcal{T}$ is broken. In the following, we would show that Eq. (S22) and Eqs. (S24, S28) are equivalent in a $\mathcal{T}$-conserved system. Specifically, under LPL, one should consider the symmetric real part of Eq. (S22), which is equivalent to the shift current as in Eqs. (S24), while under CPL, one should consider the asymmetric imaginary part of Eq. (S22), which corresponds to the injection current as in Eqs. (S28).

In order to show the equivalence, the first step is to factorize the denominator of Eq. (S22) with Sokhotski-Plemelj Formula. That is, in the limit of $\tau \to \infty$ ($\delta = 1/\tau \to 0$), one has[1]

$$
\begin{aligned}
D_1 &= \frac{1}{\omega_{mn} + i\delta} = \frac{P}{\omega_{mn}} - i\pi\delta(\omega_{mn}) \\
D_2 &= \frac{1}{\omega_{ml} - \Omega + i\delta} = \frac{P}{\omega_{ml} - \Omega} - i\pi\delta(\omega_{ml} - \Omega)
\end{aligned}
\tag{S30}
$$

here $\delta(x)$ is the Dirac delta function and P stands for the Cauchy principal value in $\boldsymbol{k}$ integration.

The next step is to rearrange the numerator of Eq. (S22), which is $N^{0abd}(\boldsymbol{k}) = v^a(\boldsymbol{k})v^b(\boldsymbol{k})v^c(\boldsymbol{k})$, according to their behavior under $\mathcal{T}$ operation. As discussed in the main text, one has $\mathcal{T}N^{0abd}(\boldsymbol{k}) = -N^{0abd*}(-\boldsymbol{k})$. Since the denominator is invariant under $\mathcal{T}$, one has $\frac{N^{0abd}(\boldsymbol{k})}{D_1(\boldsymbol{k})D_2(\boldsymbol{k})} = -\frac{N^{0abd*}(-\boldsymbol{k})}{D_1(-\boldsymbol{k})D_2(-\boldsymbol{k})}$. Consequently, after a summation over $\pm\boldsymbol{k}$, only the imaginary part of $N^{0abd}(\boldsymbol{k})$ would contribute to the final result, and thus we can ignore the real part of $N^{0abd}(\boldsymbol{k})$ and treat it as a purely imaginary quantity.

Reproduce the shift current. First, we note that the a dc current should be a real quantity.

[1]Here we focus on the first term in the bracket of Eq. (S22), the second term can be analyzed in the same fashion.

For a LPL, E^b and E^c has no phase difference and only the real part of the conductivity σ^a_{bc} contributes to the NLO current. As discussed above, the numerator $N^{0abd}(\boldsymbol{k})$ can be treated as a purely imaginary quantity, thus one needs to consider the imaginary part of the denominator, which is

$$\mathrm{Im}(D_1 D_2) = \pi \frac{P}{\omega_{mn}} \delta(\omega_{ml} - \Omega) + \pi \frac{P}{\omega_{ml} - \hbar\Omega} \delta(\omega_{mn}) \tag{S31}$$

The second term in symmetric with respect to the permutation of m, n (after summation over m, n), while the imaginary part of $v^a_{nl} v^b_{lm} v^c_{mn}$ is asymmetric with respect to m, n. Therefore only the first term in Eq. (S31) would not vanish in the final result. Now it's straightforward to check that that the integrand in Eq. (S22) is terms with format

$$\sum_{mnl} \frac{f_{lm} v^b_{lm}}{\omega^2_{ml}} \left(\frac{v^a_{mn} v^c_{nl}}{\omega_{mn}} - \frac{v^c_{mn} v^a_{nl}}{\omega_{nl}} \right) \delta(\omega_{ml} - \omega) \tag{S32}$$

where we have replaced ω in the prefactor of Eq. (S22) with ω_{ml} because of the the delta function.

On the other hand, the integrand of Eq. (S24) is

$$\sum_{ml} f_{lm} (r^b_{ml} r^c_{lm;a} + r^c_{ml} r^b_{lm;a}) \delta(\omega_{ml} - \omega) \tag{S33}$$

The equivalence between Eq. (S32) and Eq. (S33) can be proved by using Eq. (S26) and plugging in the *sum rule* for $r^b_{lm;a}$ [8]

$$r^b_{lm;a} = \frac{r^a_{lm} \Delta^b_{ml} + r^b_{lm} \Delta^a_{ml}}{\omega_{lm}} + \frac{i}{\omega_{lm}} \sum_n (\omega_{nm} r^a_{ln} r^b_{nm} - \omega_{ln} r^b_{ln} r^a_{nm}) \tag{S34}$$

Note that the first term in Eq. (S34), $F^b_{lm;a} = \frac{r^a_{lm} \Delta^b_{ml} + r^b_{lm} \Delta^a_{ml}}{\omega_{lm}}$, does not contribute to the final result because $f_{lm} r^c_{ml} F^b_{lm;a}$ is asymmetric in m, l.

Reproduce the injection current. For a circularly polarized light, E^b and E^c should have a phase difference of i. In order to get a real current, we need to consider the imaginary part of σ^a_{bc}. In this case we need to examine the real part of the denominator of Eq. (S22), which is

$$\mathrm{Re}(D_1 D_2) = \frac{P}{\omega_{mn}(\omega_{ml} - \Omega)} - \pi^2 \delta(\omega_{mn}) \delta(\omega_{ml} - \Omega) \tag{S35}$$

One can see that the first and second term in Eq. (S35) corresponds to $m \neq n$ and $m = n$, respectively. In case that $\tau \to 0$, the contribution from the first term is much smaller than the second term and thus we only consider the second term.

Putting $m = n$ ($n = l$) in the first (second) term of Eq. (S22), one has the integrand as

$$\begin{aligned}
& -i\tau \sum_{ml} \frac{f_{lm} v^b_{lm} v^c_{ml}}{\omega^2_{ml}} \left(v^a_{mm} - v^a_{ll}\right) \delta(\omega_{ml} - \omega) \\
&= i\tau \sum_{ml} f_{lm} \left(v^a_{mm} - v^a_{ll}\right) r^b_{lm} r^c_{ml} \delta(\omega_{ml} - \omega) \\
&\to \frac{i\tau}{2} \sum_{ml} f_{lm} \Delta^a_{ml} \left[r^b_{lm}, r^c_{ml}\right] \delta(\omega_{ml} - \omega)
\end{aligned} \tag{S36}$$

In the last step we have taken the asymmetry part ($bc \leftrightarrow -cb$) of the integrand. One can see that Eq. (S36) is the same as the integrand of Eq. (S28) plus a τ factor, as expected.

3 Shift- and injection-like mechanisms for the spin current

As discussed in Sec. 2, the well-known shift and injection formulae in a $\mathcal{T}$-conserved system can be reproduced from the charge current part ($j^{a,s^0} = ev^a$) of Eq. (S21). In this section, we would show that for the spin current with $j^{a,s^i} = \frac{1}{2}\{v^a, s^i\}$, $i \neq 0$, Eq. (S21) can also be broken down into *shift*-like and *injection*-like parts. By shift- (injection-) like, we mean that the conductivity scale with τ^0 (τ^1), where τ is the carrier relaxation time.

3.1 $\mathcal{P}$-broken, $\mathcal{T}$-conserved systems

We first study a $\mathcal{T}$-conserved system. As in Sec. 2, we still need to factorize the denominator of Eq. (S21) with Eq. (S30), and the arrange the numerator according to its behavior under $\mathcal{T}$ operation. Unlike $N^{0abd}(\boldsymbol{k})$, for the spin current, one has $\mathcal{T} N^{iabd}(\boldsymbol{k}) = N^{iabd*}(-\boldsymbol{k})$. Consequently, after a summation over $\pm\boldsymbol{k}$, only the *real* part of $N^{iabd}(\boldsymbol{k})$ contributes to the total conductivity, in contrast to the charge current, where the *imaginary* part of $N^{0abd}(\boldsymbol{k})$ contributes.

Linearly polarized light. Under a LPL, for the spin current one needs to consider the real part of the denominator, as in Eq. (S35). And similar algebra to that in Eq. (S36) leads to a integrand for the NLO spin conductivity as

$$\begin{aligned}
& i\tau \sum_{ml} f_{lm} \left(j^{a,s^i}_{mm} - j^{a,s^i}_{ll}\right) r^b_{lm} r^c_{ml} \delta(\omega_{ml} - \omega) \\
&\to \frac{i\tau}{2} \sum_{ml} f_{lm} \left(j^{a,s^i}_{mm} - j^{a,s^i}_{ll}\right) \left\{r^b_{lm}, r^c_{ml}\right\} \delta(\omega_{ml} - \omega)
\end{aligned} \tag{S37}$$

where we have taken the symmetry part ($bc \leftrightarrow cb$) as $\{r^b_{lm}, r^c_{ml}\} = r^b_{lm}r^c_{ml} + r^c_{lm}r^b_{ml}$ because we are dealing with LPL. One can see that for a $\mathcal{T}$-conserved system under LPL, the mechanism for spin current generation in actually *injection*-like. A prominent feature is that the NLO spin conductivity is (approximately) proportional to the lifetime τ.

Circularly polarized light. Under LPL, one needs to keep the imaginary part of the denominator as in Eq. (S31). This time the second term in Eq. (S31) would also contribute to the final result because the real part of $j^{a,s^i}_{nl} v^b_{lm} v^c_{mn}$ is also symmetric in m, n. As a result, under CPL, the contribution to the spin current has a *shift*-like part,

$$\sum_{mnl} \frac{f_{lm} v^b_{lm}}{\omega^2_{ml}} \left(\frac{j^{a,s^i}_{mn} v^c_{nl}}{\omega_{mn}} - \frac{v^c_{mn} j^{a,s^i}_{nl}}{\omega_{nl}} \right) \delta(\omega_{ml} - \omega) \tag{S38}$$

which is independent of τ, plus a *injection*-like part

$$-i\tau \sum_{ml} \frac{f_{lm} v^b_{lm} v^c_{ml}}{(\omega_{ml} - \omega)\omega^2} \left(j^{a,s^i}_{mm} - j^{a,s^i}_{ll} \right) \tag{S39}$$

which depends linearly on τ.

3.2 $\mathcal{P}$-broken, $\mathcal{T}$-broken, $\mathcal{PT}$-conserved systems

For a $\mathcal{PT}$-conserved system, one needs to study the imaginary part of $N^{iabd}(\boldsymbol{k})$ because $\mathcal{PT}N^{iabd}(\boldsymbol{k}) = -\tilde{N}^{iabd*}(-\boldsymbol{k})$. With similar analysis as in the previous section for $\mathcal{T}$-conserved system, one can find that the generation of spin current under LPL (CPL) has shift (injection)-like mechanism, respectively.

The same analysis also applies to charge current in a $\mathcal{PT}$-conserved system, and the results are listed in Table 2 of the main text.

3.3 $\mathcal{P}$-broken, $\mathcal{T}$-broken, $\mathcal{PT}$-broken system

In this kind of systems, both the real and imaginary parts of $N^{iabd}(\boldsymbol{k})$ can contribute to the conductivity for spin and charge currents. As a result, under either LPL or CPL, the shift- and injection-like mechanisms would both contribute.

4 The definition of spin current

There are lots of debates on the definition of the spin curren. In the main text we adopted the conventional definition $j_1 = \frac{1}{2}(vs + sv)$. But this definition is not entirely correct, although it is convenient, physically appealing, and extensively employed in many works until today. For example, this spin current is not conserved. Also, as suggested in Rashba's work [9], this definition would lead to a non-zero spin current even if an inversion asymmetric insulator is in equilibrium (under no electric field, light, etc.). There are lots of debates, and there are also works claiming that we do not need to modify this definition. See e.g., Ref. [10].

In Ref. [11], the authors proposed another definition, which is $j_2 = \frac{d(rs)}{dt}$. This definition can be conserved in some systems. However, it requires that "spin generation in the bulk is absent due to symmetry reasons". In other words, it requires the bulk integration $\frac{1}{V}\int dV\mathcal{T}(r) = 0$, where $\mathcal{T}(r)$ is the torque density on the spins. Unfortunately, this is not true under external light, which is necessary for our bulk spin photovoltaic effect. An intuitive picture is, under a circularly polarized light, the angular momentum of the photons can be transferred into the electron system, which obviously leads to $\frac{1}{V}\int dV\mathcal{T}(r) \neq 0$. Therefore, the definition of $j_2 = \frac{d(rs)}{dt}$ is also not perfectly correct if the system is under light illumination. Particularly, under strong light, the non-conservation of $j_2 = \frac{d(rs)}{dt}$ might be high. On the other hand, the calculation of the spin current with $j_2 = \frac{d(rs)}{dt}$ is rather involved in practice (although this definition looks simple). To the best of our knowledge, this definition has only been applied in simple model systems, and to the linear order response.

Here we roughly estimate the difference between the spin current defined with $j_1 = \frac{1}{2}(vs + sv)$ and $j_2 = \frac{d(rs)}{dt}$. Compared with j_1, j_2 has an additional term that comes from the torque on the spins [11, 12]. This term is proportional to $[H, s]$. Therefore, the relative difference $\left|\frac{j_2 - j_1}{j_1}\right|$ can be roughly estimated from $\alpha_i = \frac{\|[H,s^i]\|}{\|H\|\cdot\|s^i\|}$, where $\|\cdot\|$ indicates matrix norm. This can be naively understood in the following way. We have $j_2 = \frac{d(rs)}{dt} = \frac{dr}{dt}s + \frac{ds}{dt}r = \frac{1}{2}\{[H, r], s\} + \frac{1}{2}\{r, [H, s]\}$, where $\{a, b\} = ab + ba$ ensures hermiticity. The first term is just j_1, while the second term comes from the torque on the spins. The ratio between these two terms is (very roughly) $\alpha_i = \frac{\|[H,s^i]\|}{\|H\|\cdot\|s^i\|}$. Rigorously speaking, the position operator r needs extra care in infinite solid-state systems.

We have calculated and plotted α_z in the first Brillouin zone for MoS_2 (Figure S1a) and $MnBi_2Te_4$ (Figure S1b). One can see that α is on the order of $0.1 \sim 0.2$. From this point of view, one may deduce that the difference between j_1 and j_1 is indeed not negligible, but in general cases, it would not qualitatively change the main results.

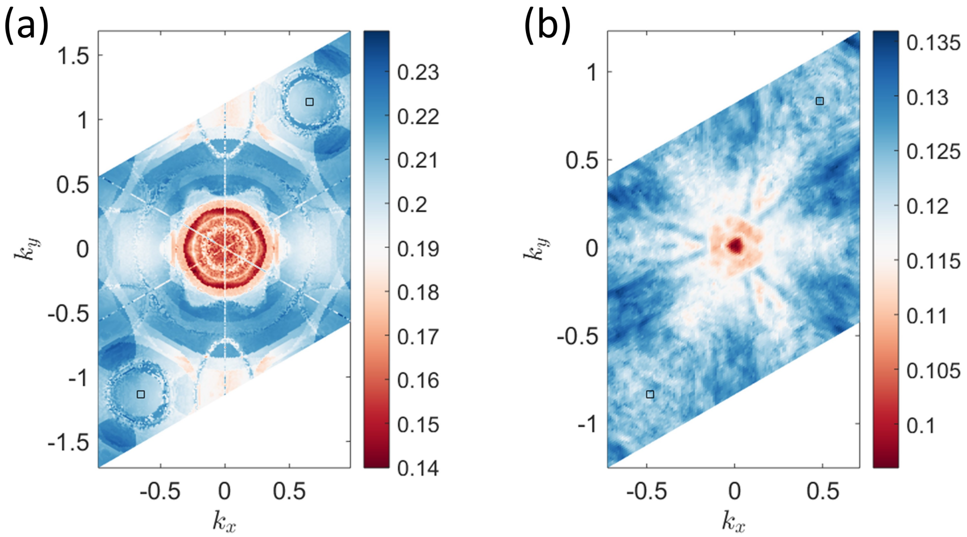

Figure S1: $\alpha_z = \frac{\|[H,s^z]\|}{\|H\|\cdot\|s^z\|}$ for (a) MoS_2 and (b) $MnBi_2Te_4$. The black boxes indicate K/K′ points in the Brillouin zone.

5 Experimental considerations

5.1 Estimation of the temperature rise

For spin current generation, the temperature rise due to the energy dissipation is generally not significant. Here we take monolayer MoS_2 as an example. For the bulk spin photovoltaic effect studied in this work, the main energy consumption is the photon absorption due to interband transitions (electron-hole pair generation), and the absorbance is $A = 1 - \exp\left[-\frac{\omega}{c\epsilon_0}\epsilon^i(\omega)d\right] \approx \frac{\sigma^r(\omega)}{c\epsilon_0}d$, where ϵ_0 is the vacuum permittivity, ϵ_i is the imaginary part of the dielectric function, σ^r is the real part of the optical conductivity. d is the thickness of the material, which is taken as 0.6 nm for MoS_2. The energy consumption rate per unit area is

$$P = AI = \frac{\sigma^r(\omega)}{c\epsilon_0}d\frac{\epsilon_0 c}{2}E^2 = \frac{\sigma^r(\omega)d}{2}E^2 \tag{S40}$$

where $I = \frac{\epsilon_0 c}{2}E^2$ is the intensity of the light. From our ab initio calculations, at $\omega = 3$ eV one has $\sigma^r(\omega) = 4 \times 10^5\ \Omega/\text{m}$. In the main text, we showed that light with electric field strength on the order of $E = 100$ V/m would be able to generate a detectable spin current. With this field strength the energy absorption power is $P = 1.2 \times 10^{-4}$ W/cm^2, which is rather small. Here we calculate the temperature rise under an electric field of $E = 1$ MV/m, which is much larger, but readily available with laser technology. At this field strength, one has $P = 1.2 \times 10^4$ W/cm^2. Assume that MoS_2 is put on a

substrate with thermal conductivity κ and thickness l_{subs}. If a continuous wave (CW) light is used, then the steady steady-state temperature rise can be roughly estimated from

$$\Delta T_{\mathrm{CW}} = \frac{P}{\kappa} l_{\mathrm{subs}} \tag{S41}$$

Assuming that $l_{\mathrm{subs}} = 1\ \mu\mathrm{m}$ and $\kappa = 100\ \mathrm{W} \cdot \mathrm{m}^{-1} \cdot \mathrm{K}^{-1}$, then $\Delta T_{\mathrm{CW}} \approx 1.2$ K, which is not significant. On the other hand, if a pulse laser is used, then the temperature rise can be estimated from

$$\Delta T_{\mathrm{pulsed}} = \frac{\tau_{\mathrm{pulse}} P S}{k_B} \tag{S42}$$

Where S is the area of a unit-cell, k_B is the Boltzmann constant, while τ_{pulse} is the duration of the pulse, and is taken as 1 ps here. One can find that $\Delta T_{\mathrm{pulsed}} \approx 0.7$ K, which is also not significant as well.

We would like to note that, not all energies absorbed by the materials goes to the phonon (lattice) system. They may be re-emitted as photons when e.g., electrons and holes recombine. Therefore, the temperature rise in the ion system may be even lower than ΔT_{CW} and $\Delta T_{\mathrm{pulsed}}$ estimated above.

5.2 Maximum spin current density

With the MoS_2 parameters, when the external field strength is $E = 1$ MV/m, the spin current density is $10^8\ \frac{\mathrm{A}}{\mathrm{m}^2} \frac{\hbar}{2e}$, and the temperature rise in the sample is estimated to be on the order of 1 K, which is not high. If we further increase the electric field strength, then two main issues will arise. 1) On the experimental side, a strong laser may destroy the sample. This might happen for electric field strength above 10 MV/m when one uses a continuous wave laser. In this case, the spin current density is around $10^{10}\ \frac{\mathrm{A}}{\mathrm{m}^2} \frac{\hbar}{2e}$, which is quite large, while the temperature rise can be as high as 100 K. Note that the temperature rise can be mitigated with better thermal management. On the other hand, the sample may survive in an even stronger electric field if the pulsed laser is used. For example, with a femtosecond laser, the electric field can be as high as 100 MV/m, with a temperature rise of 10 K. 2) On the theoretical side, the perturbation theory used in the current work may fail when the electric field is too strong. The external electric field strength should be compared with the intrinsic interaction strength in the materials, which is usually on the order of 1 V/Å $= 10^4$ MV/m. From this point of view, the perturbation theory may work up to an electric field strength of 100 MV/m. Above this strength, the error from perturbative expansions may not be ignored and non-perturbation theories may be required.

In summary, with a pulsed laser, our theoretical picture may work up to an electric field strength

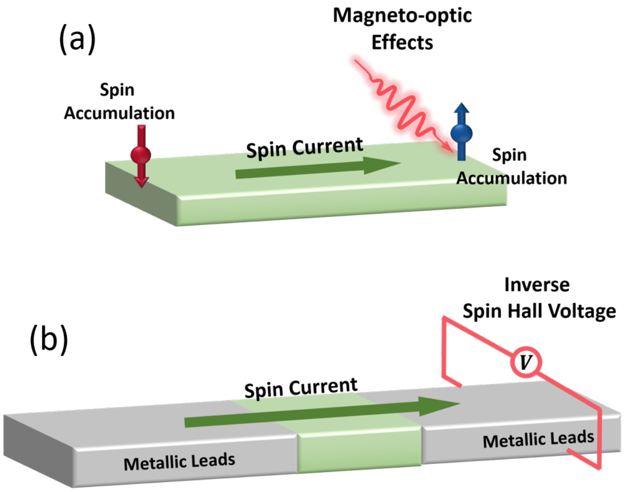

Figure S2: Approaches for detecting the spin current. (a) In a close circuit setup, there will be spin accumulations on both ends of the source material, which can be detected by magneto-optic effects. (b) In an open circuit setup, the spin current flows into metallic leads, and the inverse spin Hall voltage would be generated.

up to 100 MV/m, when the spin current density can be on the order of $10^{12}\ \frac{\mathrm{A}}{\mathrm{m}^2}\frac{\hbar}{2e}$. This is restricted by both experimental (sample damage) and theoretical (validity of perturbation theory) concerns. With a continuous wave laser, one may have to use an electric field strength below 10 MV/m to keep the temperature rise and sample damage manageable. At this field strength, the spin current density can be $10^{10}\ \frac{\mathrm{A}}{\mathrm{m}^2}\frac{\hbar}{2e}$ with the monolayer MoS_2 parameters.

5.3 Detection of the spin current

In the main text we discussed how the spin current can be detected. The schemes are illustrated in Fig. S2.

6 Computational benchmarks

In this section we provide computational benchmarks on our NLO spin/charge conductivity formula Eq. (S21).

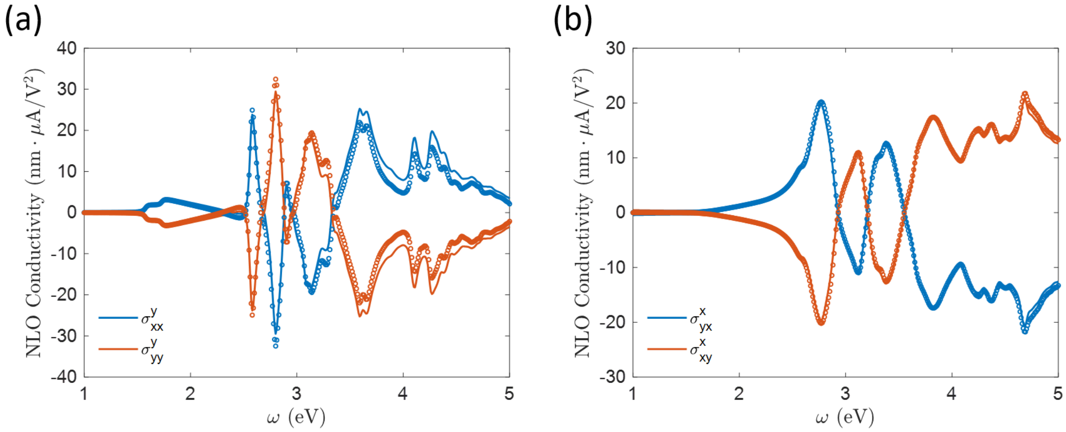

Figure S3: NLO charge conductivity of MoS_2 under LPL (a) and CPL (b). The solid lines are obtained with Eq. (S22), which uses the velocity gauge, while the dots are obtained with the well-known shift and injection current formulae Eqs. (S24, S28), which are in the length gauge. The two curves agree well with each other. There are minor discrepancies at high frequencies, and the reason is that only a finite number of conduction bands are used in the computation.

6.1 Numerical comparison between the velocity gauge and length gauge

a. We compare the charge shift and injection conductivity from Eq. (S22), which is in velocity gauge, and from Eqs. (S24, S28), which is from length gauge. We take MoS_2 as an example and the results are shown in Fig. S3. One can see that the two curves agree well with each other. Note there are minor discrepancies at high frequency ($\omega > 3.5$ eV), this is because we only use a finite number of conduction bands in the calculation. The discrepancies should disappear when infinite number of conduction bands are involved.

b. We reproduce the NLO charge conductivity for monolayer GeS, which was studied in Ref. [13]. Our results are shown in Fig. S4 . One can see that it is in great agreement with Fig. 2 in Ref. [13].

6.2 NLO conductivity as a function of lifetime τ

The analysis in Sec. 3 demonstrates that under LPL, the charge (spin) currents of MoS_2 are shift (injection) like, while the charge (spin) currents of MBT are injection (shift) like (see also Table 2 in the main text). For shift (injection) like mechanism, the conductivity scales as τ^0 (τ^1). We have numerically tested the τ-dependence for MoS_2 and MBT. The results can be found in Figs. S5 and S6, respectively. The expected dependence on τ can be clearly observed.

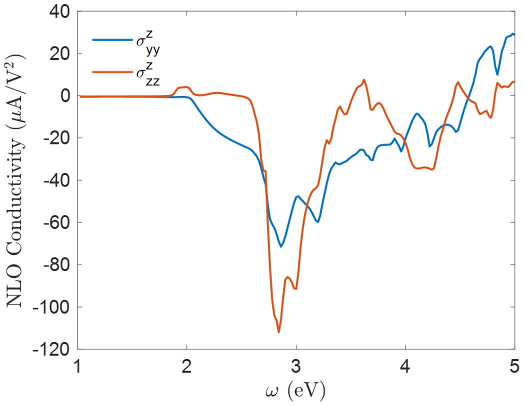

Figure S4: The NLO charge conductivity of monolayer GeS obtained with Eq. (S22), in agreement with results in Ref. [13] (Fig. 2 therein).

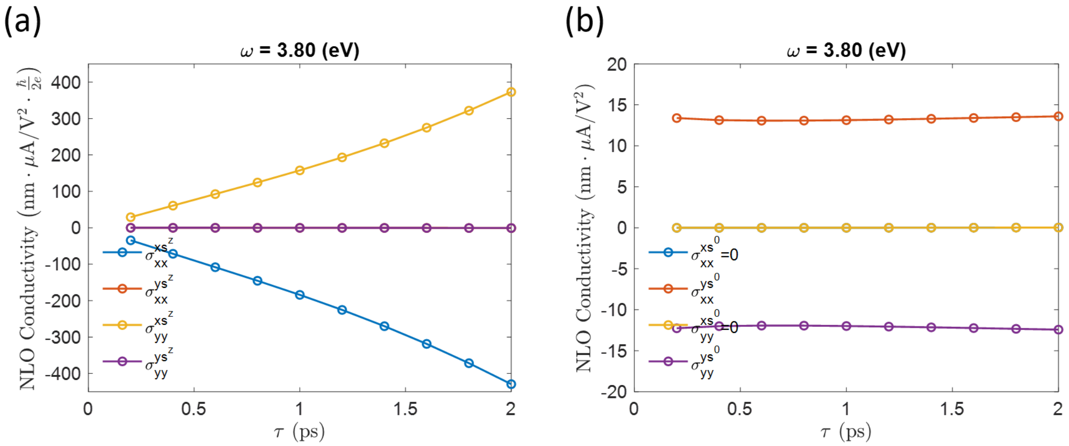

Figure S5: NLO spin (a) and charge (b) conductivity of MoS_2 under LPL as a function of carrier relaxation time τ. The spin conductivity is approximately linearly dependent on τ, while the charge conductivity remains a constant. Such behavior manifests that in a $\mathcal{T}$-symmetric system, the charge current comes from shift-like mechanism, while the spin current comes from injection-like mechanism under LPL. The conductivities are at $\omega = 3.8$ eV.

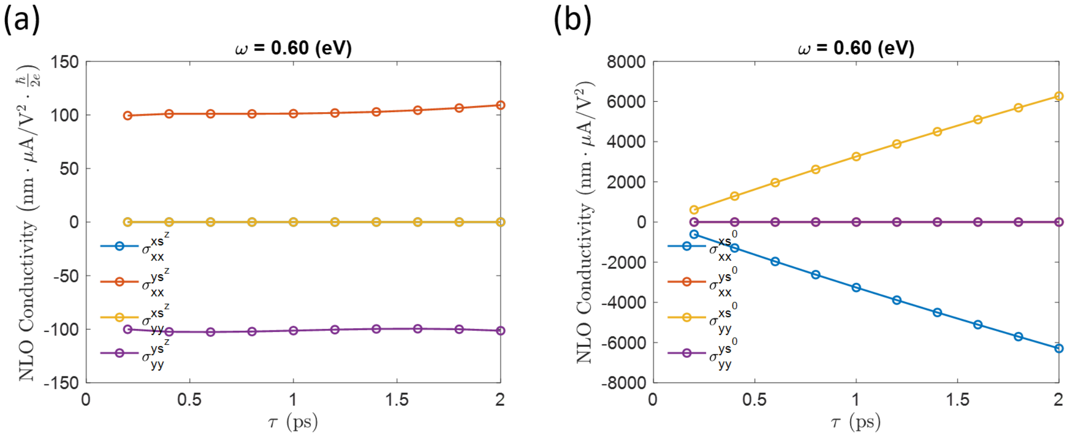

Figure S6: NLO spin (a) and charge (b) conductivity of MBT under LPL as a function of carrier relaxation time τ. The spin conductivity is approximately independent of τ, while the charge conductivity is approximately linearly dependent on τ. Such behavior manifests that in a $\mathcal{PT}$-symmetric system, the spin current comes from shift-like mechanism, while the charge current comes from the injection-like mechanism under LPL. The conductivities are at $\omega = 0.6$ eV.

6.3 NLO conductivity as a function of SOC strength

In the main text, we argued that in MBT, the NLO charge conductivity should increase with the SOC strength λ. This is computationally verified in Fig. S7.

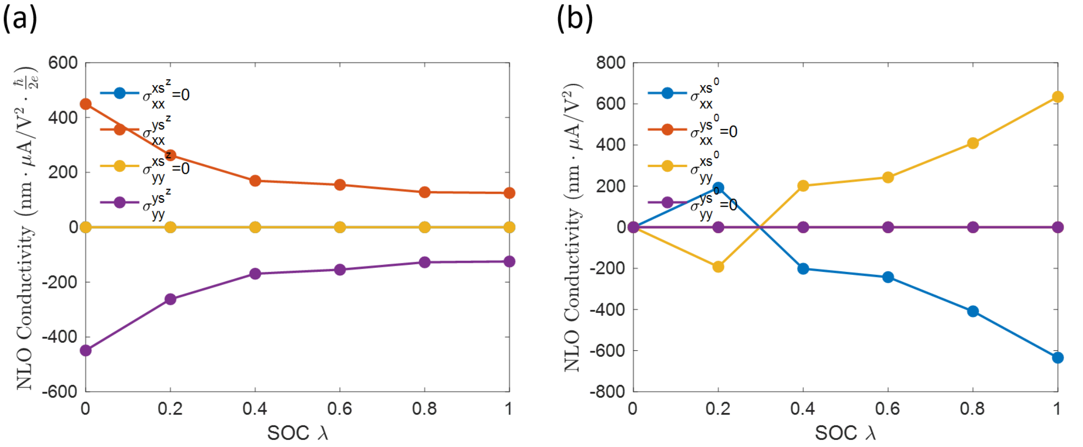

Figure S7: Peak values of NLO spin (a) and charge (b) conductivity of MBT as a function of SOC strength. As discussed in the main text, the spin conductivity is nonzero even when SOC is completely turned off ($\lambda = 0$), while the charge conductivity is zero when $\lambda = 0$ due to inversion-spin rotation symmetry and grows with λ. Note that SOC strongly modifies the band structure of MBT, thus the spin and charge conductivity have relatively more complicated dependence on λ, as compared that in the case of MoS_2.

7 NLO spin current under circularly polarized light

In the main text, the NLO spin currents under LPL are presented. They correspond to the symmetric real part of Eq. (S21). In this section we show the spin current under CPL as in Figs. S8-S10, which correspond to the asymmetric imaginary part of Eq. (S21).

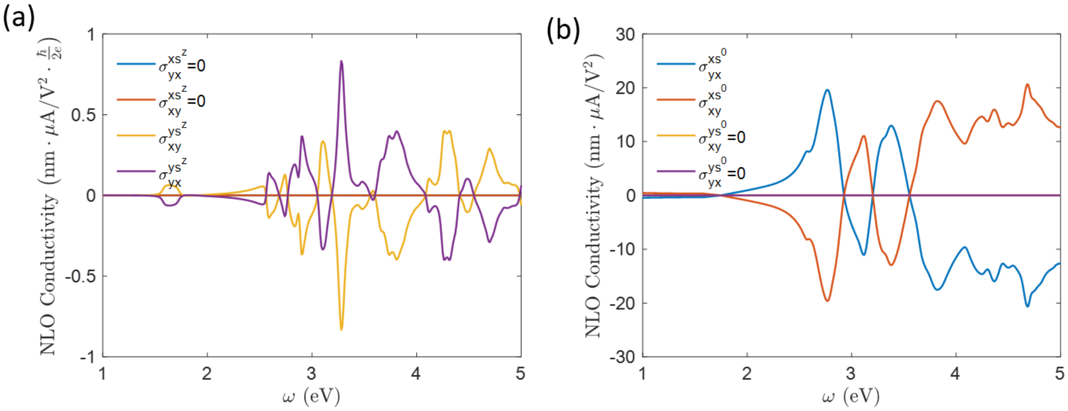

Figure S8: The NLO charge and spin-z conductivity of MoS_2 under CPL.

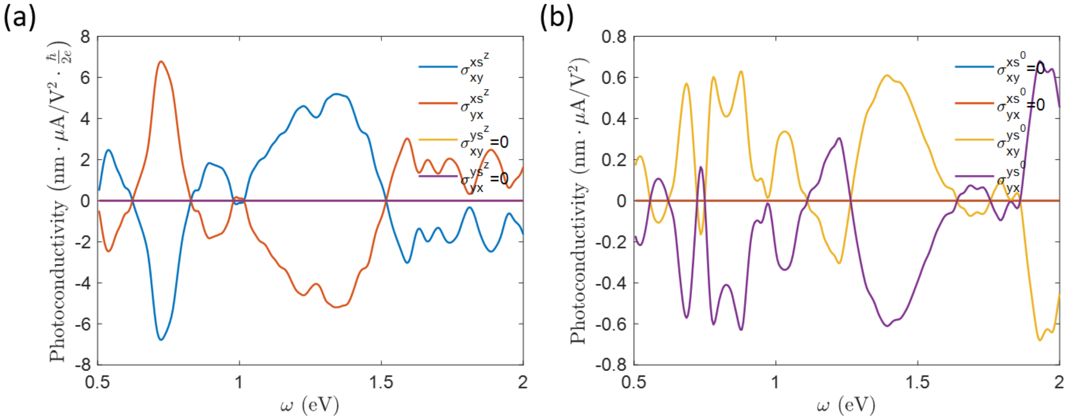

Figure S9: The NLO charge and spin-z conductivity of $MnBi_2Te_4$ under CPL.

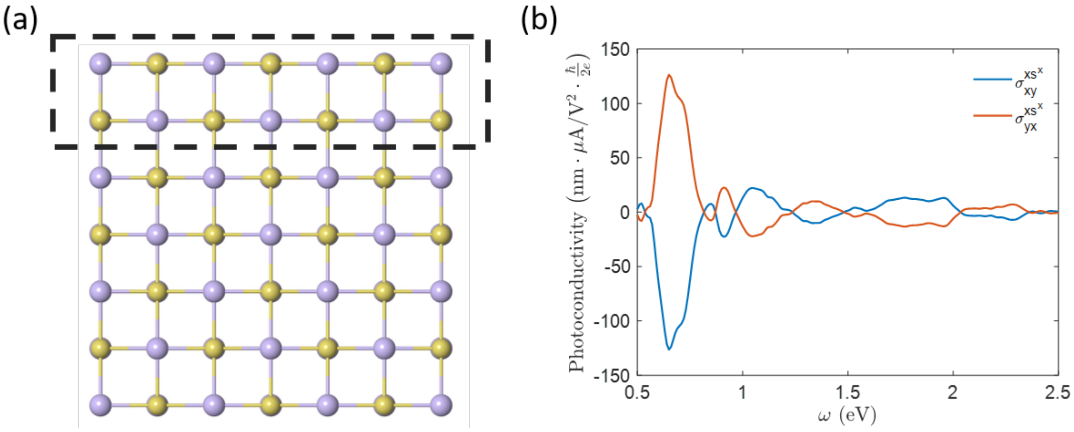

Figure S10: (a) Side view of SnTe. The dashed box indicate the out-most layer of SnTe. (b) The NLO spin conductivity of SnTe under CPL.

References

[1] Kraut, W. & von Baltz, R. Anomalous bulk photovoltaic effect in ferroelectrics: a quadratic response theory. *Physical Review B* **19**, 1548 (1979).

[2] von Baltz, R. & Kraut, W. Theory of the bulk photovoltaic effect in pure crystals. *Physical Review B* **23**, 5590 (1981).

[3] Zhang, Y. *et al.* Photogalvanic effect in weyl semimetals from first principles. *Physical Review B* **97**, 241118 (2018).

[4] Zhang, Y. *et al.* Switchable magnetic bulk photovoltaic effect in the two-dimensional magnet cri 3. *Nature communications* **10**, 1–7 (2019).

[5] Sipe, J. & Shkrebtii, A. Second-order optical response in semiconductors. *Physical Review B* **61**, 5337 (2000).

[6] Ventura, G., Passos, D., dos Santos, J. L., Lopes, J. V. P. & Peres, N. Gauge covariances and nonlinear optical responses. *Physical Review B* **96**, 035431 (2017).

[7] Taghizadeh, A., Hipólito, F. & Pedersen, T. G. Linear and nonlinear optical response of crystals using length and velocity gauges: Effect of basis truncation. *Physical Review B* **96**, 195413 (2017).

[8] Aversa, C. & Sipe, J. Nonlinear optical susceptibilities of semiconductors: Results with a length-gauge analysis. *Physical Review B* **52**, 14636 (1995).

[9] Rashba, E. I. Spin currents in thermodynamic equilibrium: The challenge of discerning transport currents. *Physical Review B* **68**, 241315 (2003).

[10] Sun, Q.-f., Xie, X. & Wang, J. Persistent spin current in nanodevices and definition of the spin current. *Physical Review B* **77**, 035327 (2008).

[11] Shi, J., Zhang, P., Xiao, D. & Niu, Q. Proper definition of spin current in spin-orbit coupled systems. *Physical review letters* **96**, 076604 (2006).

[12] Murakami, S. Quantum spin hall effect and enhanced magnetic response by spin-orbit coupling. *Physical Review Letters* **97**, 236805 (2006).

[13] Rangel, T. *et al.* Large bulk photovoltaic effect and spontaneous polarization of single-layer monochalcogenides. *Physical review letters* **119**, 067402 (2017).
